# Role of Machine Learning (ML)-Based Classification Using Conventional ^18^F-FDG PET Parameters in Predicting Postsurgical Features of Endometrial Cancer Aggressiveness

**DOI:** 10.3390/cancers15010325

**Published:** 2023-01-03

**Authors:** Carolina Bezzi, Alice Bergamini, Gregory Mathoux, Samuele Ghezzo, Lavinia Monaco, Giorgio Candotti, Federico Fallanca, Ana Maria Samanes Gajate, Emanuela Rabaiotti, Raffaella Cioffi, Luca Bocciolone, Luigi Gianolli, GianLuca Taccagni, Massimo Candiani, Giorgia Mangili, Paola Mapelli, Maria Picchio

**Affiliations:** 1 Faculty of Medicine and Surgery, Vita-Salute San Raffaele University, Via Olgettina 58, 20132 Milan, Italy; 2 Nuclear Medicine Department, IRCCS San Raffaele Scientific Institute, Via Olgettina 60, 20132 Milan, Italy; 3 Unit of Obstetrics and Gynaecology, IRCCS San Raffaele Scientific Institute, Via Olgettina 60, 20132 Milan, Italy; 4 School of Medicine and Surgery, University of Milano-Bicocca, Piazza dell’Ateneo Nuovo 1, 20126 Milan, Italy,; 5 Pathology Unit, IRCCS San Raffaele Scientific Institute, Via Olgettina 60, 20132 Milan, Italy

**Keywords:** endometrial cancer, ^18^F-FDG PET, machine learning, prognostic value, imaging parameters

## Abstract

**Simple Summary:**

Early and accurate assessment of endometrial cancer (EC) aggressiveness is of utmost importance for correct treatment in affected patients. However, features of EC aggressiveness are currently assessable only after surgery. The aim of the present study was to investigate the role of machine learning (ML)-based classification using ^18^F-FDG PET parameters in preoperatively characterizing and predicting features of EC aggressiveness. Precisely, a signature integrating the most conventional PET parameters and clinical data was built. As a result, the described approach allowed the characterization and prediction of the investigated features of EC aggressiveness, demonstrating how advanced PET image analysis based on conventional quantitative parameters and ML can complement qualitative analysis, supporting the non-invasive preoperative stratification and treatment management of EC patients, in an interpretable and applicable way.

**Abstract:**

Purpose: to investigate the preoperative role of ML-based classification using conventional ^18^F-FDG PET parameters and clinical data in predicting features of EC aggressiveness. Methods: retrospective study, including 123 EC patients who underwent ^18^F-FDG PET (2009–2021) for preoperative staging. Maximum standardized uptake value (SUVmax), SUVmean, metabolic tumour volume (MTV), and total lesion glycolysis (TLG) were computed on the primary tumour. Age and BMI were collected. Histotype, myometrial invasion (MI), risk group, lymph-nodal involvement (LN), and *p53* expression were retrieved from histology. The population was split into a train and a validation set (80–20%). The train set was used to select relevant parameters (Mann-Whitney U test; ROC analysis) and implement ML models, while the validation set was used to test prediction abilities. Results: on the validation set, the best accuracies obtained with individual parameters and ML were: 61% (TLG) and 87% (ML) for MI; 71% (SUVmax) and 79% (ML) for risk groups; 72% (TLG) and 83% (ML) for LN; 45% (SUVmax; SUVmean) and 73% (ML) for *p53* expression. Conclusions: ML-based classification using conventional ^18^F-FDG PET parameters and clinical data demonstrated ability to characterize the investigated features of EC aggressiveness, providing a non-invasive way to support preoperative stratification of EC patients.

## 1. Introduction

Endometrial cancer (EC) is the most common gynecological malignancy in high- and middle-income countries [1]. Risk of EC is positively correlated with obesity, older age, early menarche, and late menopause [2,3]. Based on histology, EC is classified into endometrioid tumours, which account for 70–80% of all endometrial cancers [4] and non-endometrioid tumours (10–20%), which include serous, clear-cell, mixed cell adenocarcinomas, and other relatively rare types of tumours with poor prognosis [5].

For the most optimal treatment planning, an early and accurate assessment of EC status and aggressiveness is of utmost importance. EC treatment includes surgery, radiation, standard chemotherapy, and hormonal treatment [1]. Moreover, immune checkpoint inhibitors and VEGF inhibitors have shown encouraging results in patients with advanced endometrial carcinoma in terms of efficacy and safety profiles [6].

Radical surgery and lymphadenectomy are generally recommended for high risk patients. However, the correct selection of patients who might benefit from this kind of treatment is still challenging [7] and, especially when dealing with young patients of reproductive age, fertility sparing approaches need to be considered [8,9,10]. International Federation of Obstetrics and Gynecology (FIGO) stage, histology, depth of myometrial invasion (MI) [7], and lymph node (LN) metastases are the most commonly reported features of EC aggressiveness [11,12,13]. Moreover, recent findings demonstrate that genomic features may strongly influence EC behaviour and prognosis, and the molecular characterization of EC subtypes has become part of the risk stratification of disease [14,15]. In particular, *p53* overexpression is recognized as a relevant prognostic factor in EC, being also involved in the regulation of several genetic factors including PTEN, which has been shown to be associated with unfavourable prognosis in various types of cancer [5,16,17,18].

As a limit, many of these features of aggressiveness can only be assessed after surgery, with few of them assessable on bioptic samples. Biopsy, however, may not represent the whole tumour heterogeneity and therefore may provide only limited information on tumour aggressiveness before surgical treatment [19,20]. Other strategies to characterize tumour behavior include genomic and proteomic analysis, which have improved patient outcome by uncovering genetic and molecular signaling affecting therapeutic efficacy [21].

Conventional imaging modalities, including transvaginal ultrasound, magnetic resonance imaging (MRI), and computed tomography (CT), provide detailed morphological information on EC, with only limited assessment of functional characteristics [22,23]. ^18^F-fluoro-deoxyglucose (^18^F-FDG) positron emission tomography (PET) has a well-established role in the preoperative staging of EC patients, and it is included in the clinical guidelines on EC management [24,25]. Precisely, ^18^F-FDG is useful in providing a whole-body assessment of the disease, therefore identifying possible LN involvement and distant metastases [26,27,28,29,30].

Medical images are commonly evaluated with qualitative analysis by expert physicians. However, the estimation of quantitative data such as imaging-derived parameters has recently attracted great interest [31] and is currently under evaluation as an innovative tool for improving disease characterization and tumour heterogeneity [32]. With respect to ^18^F-FDG PET images, standardized uptake value maximum and mean (SUVmax, SUVmean), metabolic tumour volume (MTV), and total lesion glycolysis (TLG) are among the most encountered and investigated parameters, now recognized as biomarkers of pathophysiological processes in several types of tumours [33,34,35,36]. Moreover, compared to the earliest radiomic features currently under investigation, these parameters are not limited by clinical applicability and interpretability, being: (i) easily computable by physicians using standard clinical software during a conventional qualitative analysis, (ii) clearly interpretable by clinicians as strictly correlated to biological tumour processes. Contrarily, these aspects represent two of the main limitations when dealing with radiomic features, challenging the methodology by emerging from a research topic as a useful tool in clinical settings.

At the same time, machine learning (ML) is emerging in clinical research as a powerful analytical approach aimed at supporting the clinical decision-making process [37]. Learning from past patients’ records, ML models are able to predict future outcomes, such as features of tumour aggressiveness currently assessable only after surgery, therefore supporting more accurate stratification and treatment planning for patients.

In the present study, the role of machine learning models in preoperatively predicting several features of EC aggressiveness will be investigated. Precisely, conventional ^18^F-FDG PET parameters, including SUVmax, SUVmean, MTV, and TLG, will be first individually investigated and then combined as ML inputs together with standard clinical data, aiming at supporting the most optimal EC stratification and treatment planning, in the most clinically interpretable and applicable way.

## 2. Materials and Methods

### 2.1. Patients

In this retrospective monocentric study, all consecutive patients with biopsy proven EC who underwent to ^18^F-FDG PET at the Nuclear Medicine Department of IRCCS San Raffaele Scientific Institute from August 2009 to February 2021 for staging purpose were included. Inclusion criteria were: (i) histological diagnosis of EC, (ii) availability of ^18^F-FDG PET scan performed for staging purpose. Exclusion criteria was the non-availability of imaging, clinical, and histological data required for the analyses.

Clinical and histological data were collected, including patients’ age and BMI, histological subtype (endometrioid vs. non-endometrioid), presence of deep MI defined as MI > 50%, EC risk group, presence of LN involvement, and expression of *p53* genetic marker (overexpressed: mutational-type; null: wild-type).

Patients were divided into a low risk group (low and intermediate risk) and a high risk group (high-intermediate and high risk), according to the ESMO-ESGO-ESTRO consensus conference classification [24].

Due to the multiple predictions performed in the study regarding the different features of EC aggressiveness, different cohorts were generated based on the availability of specific histological data.

This study was approved by the Institutional Ethics Committee of IRCCS San Raffaele Scientific Institute (138/INT/2021), and all patients gave their informed consent to participate to the study. All procedures were carried out in accordance with the Declaration of Helsinki (1964) and its later amendments.

### 2.2. ^18^F-FDG PET Protocols

Patients’ preparation, radiotracer injection, and acquisition protocol were performed as previously described [22]. In relation to the retrospective design of the study, different tomographs were used: (1) a fully hybrid 3T PET/MRI system (SIGNA PET/MRI; General Electric Healthcare, Waukesha, WI, USA), (2) a Discovery ST (General Electric Healthcare), (3) a Discovery STE (General Electric Healthcare), (4) a Gemini-GXL (Philips Medical Systems, Eindhoven, The Netherlands), and (5) a Discovery 690 (General Electric Healthcare). The PET scans were performed in 2-D mode (4 min per bed position) with the Discovery ST, while 3-D mode acquisition was used with the PET/MRI (4 min per bed position), Discovery STE (2.5 min per bed position), Discovery 690 (3 min per bed position), and Gemini GXL (2 min per bed position). PET raw data were corrected for random, scatter and attenuation, and reconstructed. To overcome the impact of PET image acquisition and reconstruction factors (scanner effects) on imaging parameters, the ComBat harmonization method and tool [38] were used.

### 2.3. ^18^F-FDG PET Qualitative and Semiquantitative Image Analysis

Images read-out was performed by two experienced Nuclear Medicine physicians on the Advanced Workstation (AW, General Electric Healthcare, Waukesha, WI, USA), allowing the visualization of PET images in axial, coronal, and sagittal planes. ^18^F-FDG PET images were qualitatively interpreted, and a consensus on each scan included in the study was reached by the readers. ^18^F-FDG uptake was considered pathological when higher compared to the physiological activity. In cases of pathological ^18^F-FDG uptake, the exact anatomic location was defined based on morphological images. Regarding semiquantitative analysis, volumes of interest (VOIs) showing pathological radiotracer uptake on the primary tumour were semi-automatically defined on transaxial PET images. Three-dimensional volumetric measurements of the following PET semiquantitative parameters were assessed: (1) SUVmax, (2) SUVmean, (3) MTV, and (4) TLG. For those PET scans not showing any ^18^F-FDG pathological uptake corresponding to the primary tumour, an arbitrary value of 0,1 was assigned to each parameter.

### 2.4. Surgery and Histopathological Analysis

One hundred twenty/123 patients underwent surgical intervention within 1 month from the ^18^F-FDG PET scan. Surgery consisted in total open or laparoscopic hysterectomy, bilateral salpingo-oophorectomy, peritoneal washing, nodal staging with pelvic/para-aortic lymphadenectomy, or sentinel lymph node (SLN) dissection. A pathologist specialized in gynecologic oncology (more than 30 years of experience), blinded to PET findings, performed histopathologic examination of all cases with multiple sections for each case. For each case, histological subtype, type of myometrial infiltration pattern, and lymph node involvement were evaluated and collected for analyses. In addition, *p53* immunohistochemical parameter was considered and collected: positivity for *p53* was correlated to mutational-type or wild-type expression (overexpressed or null). For nodal staging, histopathological findings after pelvic/para-aortic lymphadenectomy or sentinel lymph node (SLN) dissection, as well as imaging follow-up, were used as reference standards. Staging was assessed according to the FIGO classification of endometrial tumors. The 3/123 patients who did not undergo surgical intervention were only included in the analyses regarding the EC risk group and histological subtype, using biopsy as a reference standard.

### 2.5. Statistical Analysis

Statistical analyses were performed to assess the predictive role of ^18^F-FDG PET parameters and known preoperative clinical factors, such as patients’ age and BMI, with respect to tumour’s features of aggressiveness, including EC histological subtype (endometrioid vs. non-endometrioid), presence of deep MI, EC risk group (low and intermediate risk vs. high-intermediate and high risk), presence of LN involvement, and *p53* genetic marker expression (overexpressed vs. null).

The Kolmogorov-Smirnov test was used to assess the distribution of parameters’ values. For the prediction of each feature of EC aggressiveness, based on the available cohort, each population was randomly split into a training set (80%) and a validation set (20%), with stratified selection and no overlapping. On the training set, the nonparametric Mann-Whitney U test was performed. To avoid Type I errors (false positives), adjustment for multiple comparisons was performed using the Benjamini-Hochberg correction, and parameters with adjusted *p*-value < 0.05 (statistical significance) were subsequently analysed. The receiver operating characteristic (ROC) curve analysis was used to evaluate PET and clinical parameters’ performance in predicting EC features of aggressiveness; the area under the curve (AUC), along with its 95% confidence interval (CI) were used to compare parameters’ performance, and optimal cut-off was derived by choosing the value corresponding to the point on the ROC curve nearest to the upper left corner of the ROC graph (Youden Index method). For each feature of EC aggressiveness, parameters’ optimal cut-off values were used as threshold to classify patients of the validation set, and the predicted and corresponding reference-value pairs were recorded in a confusion matrix for performance evaluation. Precisely, accuracy, sensitivity, specificity, negative predictive value (NPV), and positive predictive value (PPV) were measured and compared. All statistical analyses were performed using Python 3.7 (Scotts Valley, CA, USA).

### 2.6. Machine Learning

A Machine Learning model was specifically implemented and optimized for the prediction of each feature of EC aggressiveness, including the presence of deep MI, the EC risk group, the presence of LN involvement, and the *p53* expression

With respect to the model type, a Random Forest Classifier (RFC) was chosen for all the prediction outcomes. RFCs, performing bootstrap sampling and feature sampling, are in fact not affected by multi-collinearity issues, automatically dropping redundant features at each tree split. This characteristic is particularly relevant when using conventional PET parameters as inputs into ML models, as they are commonly subject to multi-collinearity.

For each model to be implemented, one for each feature of EC aggressiveness, the following methodologies were specifically applied.

To avoid data leakage, the selection of model inputs was performed considering only the training set. Precisely, input data were selected based on their previously demonstrated prognostic value (see Section 2.5), by discarding parameters showing a non-statistically significant AUC’s 95% CI. Hyperparameters tuning was also performed on the training set exclusively. Finally, optimized models were tested on the validation set, and accuracy, sensitivity, specificity, negative predictive value (NPV), and positive predictive value (PPV) were measured. Machine learning model implementation was performed using the Scikit-learn library [39] (Python 3.7, Scotts Valley, CA, USA).

## 3. Results

### 3.1. Patients’ Population

**Figure A1:**
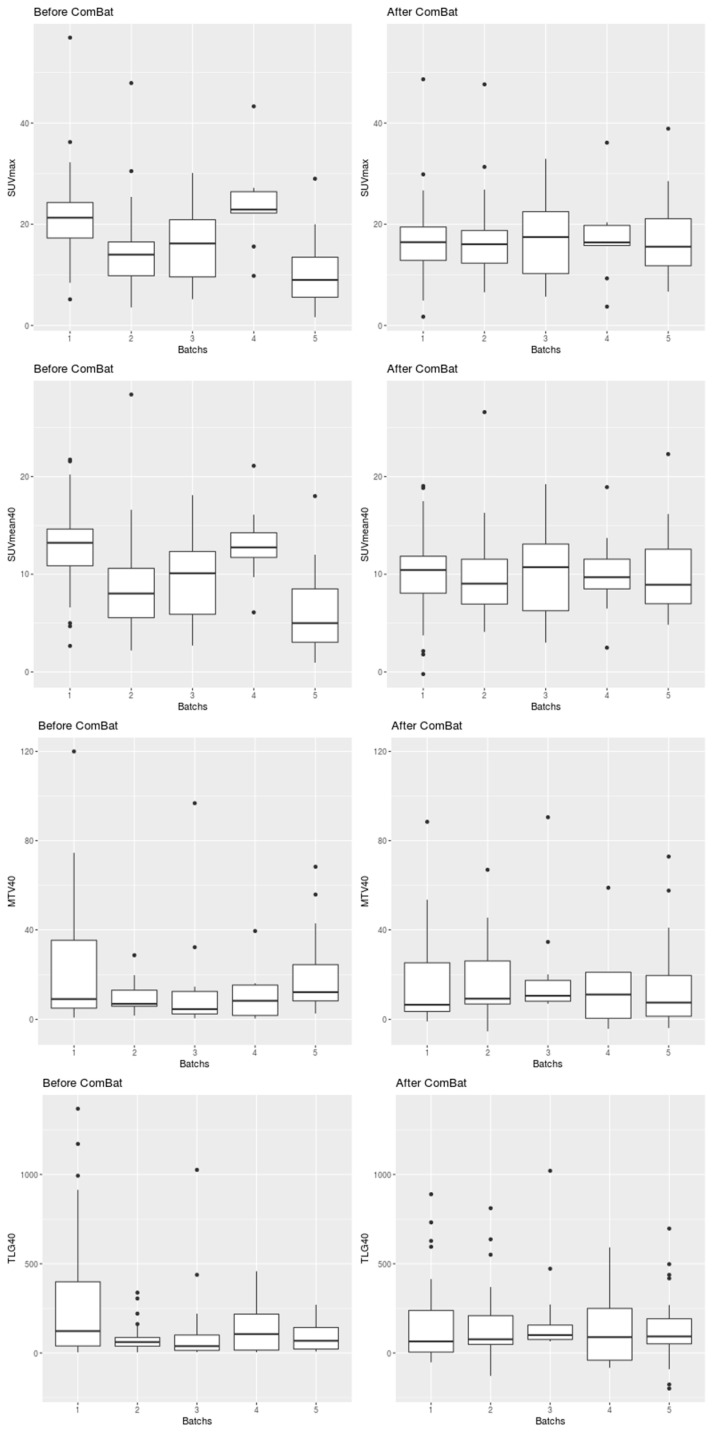
Boxplots showing distributions of PET parameters according to the different tomographs before and after ComBat harmonization. Batches: 1 = fully hybrid 3 T PET/MRI system (SIGNA PET/MRI; GE Healthcare); 2 = Discovery STE (GE Healthcare); 3 = Discovery ST (GE Healthcare); 4 = Discovery 690 (GE Healthcare); 5 = Gemini–GXL (Philips Medical Systems); SUV: standardized uptake value; MTV: metabolic tumour volume; TLG: total lesion glycolysis.

One hundred twenty-three patients with histological diagnosis of EC and availability of ^18^F-FDG PET scan performed for staging purpose were included in the study. Thirty-eight/123 patients underwent ^18^F-FDG PET/MRI and 85/123 patients underwent ^18^F-FDG PET/CT. To overcome the impact of PET image acquisition and reconstruction factors (scanner effects) on imaging parameters, the ComBat harmonization method and tool [23] were used, and harmonized PET parameters were used in subsequent analyses (Appendix A, Figure A1). Moreover, scanners’ performances were previously investigated and were assessed similar and comparable in terms of spatial resolution [40,41,42,43].

The mean age was 65 years (SD: 10.74) and the mean BMI was 27 (SD: 5.42). Due to the multiple nature of the investigation, for the analysis of each feature of EC aggressiveness different subpopulations were considered, based on the availability of histopathological data. Specifically, 85/123 patients (69.1%) presented an endometrioid histotype of EC; MI greater than 50% was present in 53/115 patients (46.1%); 76/119 patients (66.4%) were classified as high-intermediate/high risk; 37/51 patients (72.5%) had *p53* overexpression. Finally, 90 patients were considered for the analysis of LN involvement; of these, 46/90 underwent pelvic systematic lymphadenectomy, 23/90 biopsy sampling, 19/90 SLN dissection, and 2/90 patients were evaluated at defined imaging and clinical timepoints (minimum follow-up of these patients: 1-year post-surgery). Overall, LN involvement was present in 14/90 patients (15.5%).

Pathological ^18^F-FDG uptake was detected in correspondence to the primary tumour in 119/123 patients (96.7%).

Patients’ demographics and tumour characteristics are presented in Table 1.

### 3.2. Predictive PET and Clinical Parameters

**Figure 1:**
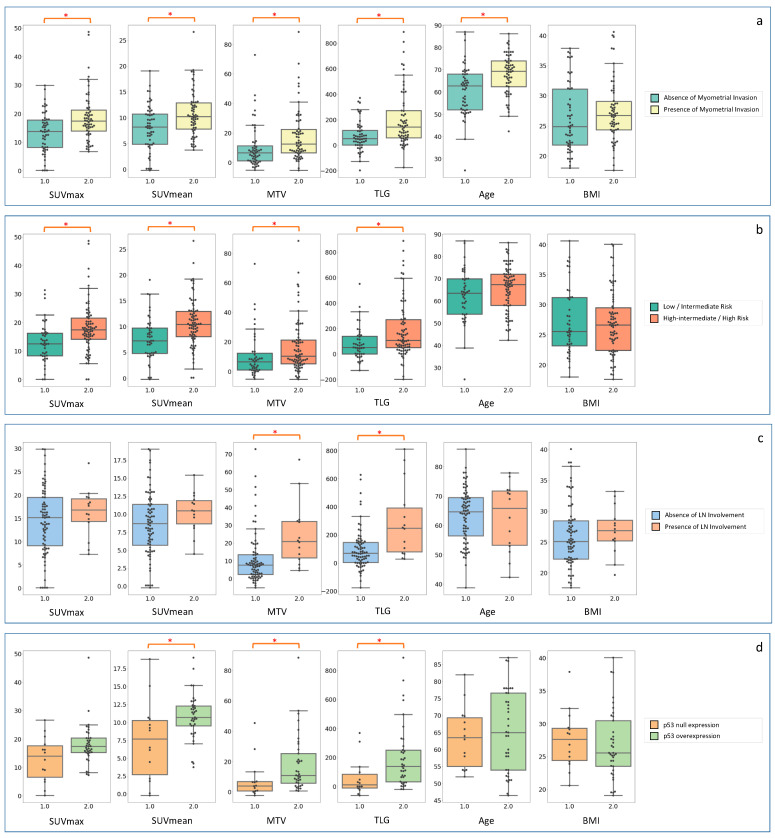
Distributions of ^18^F-FDG PET and clinical parameters’ values with respect to the different features of EC aggressiveness. Boxplots represent parameter’s distribution according to: (**a**) presence of deep myometrial invasion (>50%); (**b**) risk group classification; (**c**) presence of lymph nodes (LN) involvement; (**d**) *p53* expression. Differences that are statistically significant (Mann-Whitney U test’s adjusted *p*-value < 0.05) are marked with a red *. SUV = standardized uptake value; MTV = metabolic tumour volume; TLG = total lesion glycolysis; BMI = body mass index.

^18^F-FDG PET parameters demonstrated discriminative ability in differentiating patients according to all the investigated features of EC aggressiveness, except for the histological subtype (Table 2; Figure 1). Precisely, SUVmax and SUVmean were able to differentiate patients with respect to the presence of deep MI and the EC risk group, with SUVmean also showing a role in discriminating the *p53* expression. Moreover, MTV and TLG demonstrated their ability in discriminating deep MI, EC risk group, LN involvement and *p53* expression. Conversely, age and BMI demonstrated poor discriminative potential, with only age being able to differentiate patients with respect to the presence of deep MI.

**Figure 2:**
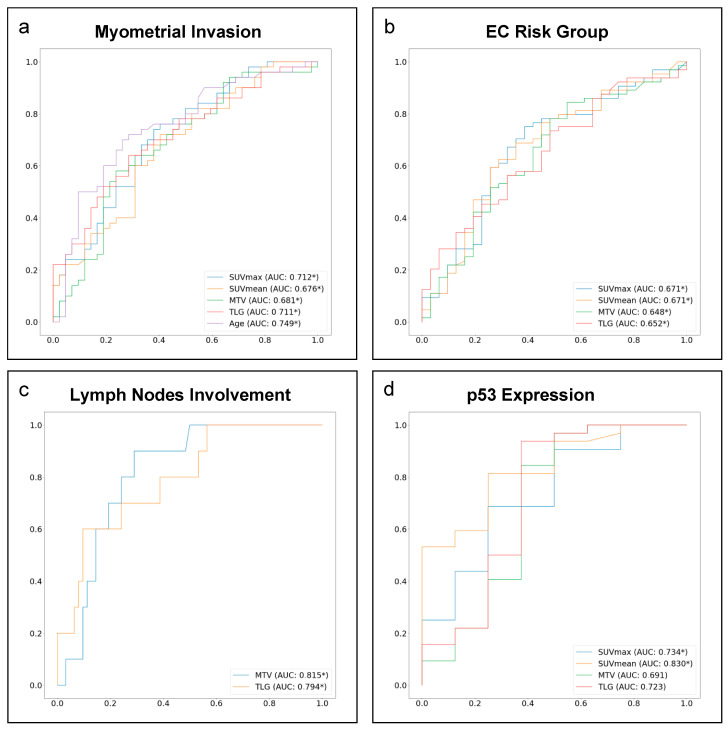
ROC curves of ^18^F–FDG PET and clinical parameters as predictors of the different features of EC aggressiveness. Solid lines represent the area under the curve (AUC) obtained for SUVmax (light blue). SUVmean (orange). MTV (green). TLG (red) and age (purple) in predicting: (**a**) presence of deep myometrial invasion (>50%); (**b**) risk group classification; (**c**) presence of lymph nodes involvement; (**d**) *p53* expression. AUC values coupled with a statistically significant 95% confidence interval are marked with *. SUV = standardized uptake value; MTV = metabolic tumour volume; TLG = total lesion glycolysis.

SUVmax and SUVmean demonstrated a role in predicting the presence of deep MI. EC risk group and the *p53* expression, while MTV and TLG showed potential in the prediction of deep MI, EC risk group, LN involvement, and *p53* expression. In contrast, no parameter was able to predict histological subtype. Finally, patients’ ages and BMI revealed a poor predictive role, with only age being predictor of deep MI. AUCs with corresponding 95% CI. optimal cut-off values and correspondent sensitivity and specificity computed on the training set are summarized in Table 3. ROC curves are displayed in Figure 2.

**Figure 3:**
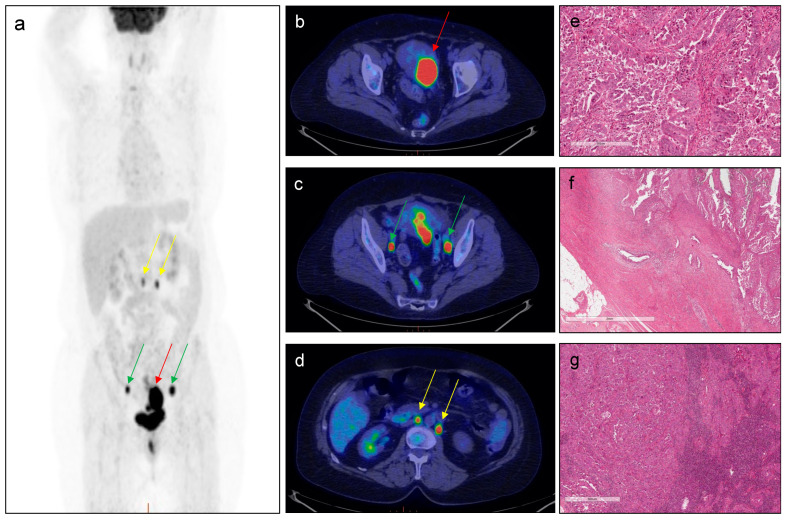
**^18^F–FDG PET performed for EC staging**. A 71-year-old patient with endometrial cancer (Stage: III C1; Grade: 3; histotype: non-endometrioid EC; MI: 85%; risk group: high–intermediate/high; presence of LNs) who underwent ^18^F–FDG PET/CT for staging purpose. Red arrows indicate pathological uptake in correspondence of the primary tumour. green arrows indicate pathological uptake in correspondence of bilateral iliac–obturator lymph nodes and yellow arrows indicate pathological uptake in correspondence of lomboaortic and interaortocaval lymph nodes ((**a**): MIP; (**b–d**): transaxial PET/CT images). PET parameters of the primary tumour were as follow: SUVmax = 17.43; SUVmean = 11.82; MTV = 28.60; TLG = 338.04. Histological analysis: the tumor is constituted by large cells with a high grade of nuclear atypia and numerous mitotic figures (**e**). The tumoral growth is mainly in papillary projections. Myometrial infiltration (**f**) has a tubulo–glandular architecture with micro–papillary structure into the lumen; the way of myometrial invasion is infiltrative/destructive. Lymph nodal metastasis (**g**) is nodular and constituted by serous atypical cells arranged in cords and small nests.

A representative case of a patient with the respective PET parameters’ values and features of EC aggressiveness is reported in Figure 3.

Accuracy. sensitivity. specificity. NPV and PPV metrices derived by using the obtained cut-offs to classify patients of the validation set are reported in Table 4. Precisely, the TLG parameter provided the best accuracy in the prediction of deep MI and LN involvement (61% and 72%. respectively), while SUVmax resulted in being the best predictor for the EC risk group (accuracy = 71%). With respect to the *p53* expression, obtained prediction performances were scarce (accuracy = 45%).

### 3.3. Machine Learning

For each feature of EC aggressiveness, an RFC (RFC_MI_. RFC_RG_. RFC_LN_. and RFC_p53_) was implemented using the training set.

Based on the AUC findings measured on the training set (see Table 3), parameters with statistically significant 95% CI were selected. Precisely, SUVmax. SUVmean. MTV, TLG, and age were used as input in the RFC_MI_; SUVmax. SUVmean. MTV and TLG were used as input in the RFC_RG_; MTV and TLG were used as input in the RFC_LN_; SUVmax, SUVmean were used as input in the RFC_p53_. For each model, bootstrap was used, with a smaller set of the training observation used to build the RF trees. To overcome class imbalance in the prediction of EC risk groups and presence of LN involvement, the “class weight” parameter was used to assign a higher weight to the minority class. Optimized hyperparameters were identified for each model and described in Table 5.

Predictions’ accuracy, sensitivity, specificity, NPV, and PPV obtained by the models when tested on the validation set are shown in Table 4. Precisely, MI was predicted with an accuracy of 87% using all PET parameters and age. Risk group was predicted with an accuracy of 79% using all PET parameters. LN involvement was predicted with an accuracy of 83% using MTV and TLG. Finally, *p53* expression was predicted with an accuracy of 73% using SUVmax and SUVmean.

## 4. Discussion

The present study demonstrates the valuable role of ML–based classification using conventional ^18^F–FDG PET parameters and clinical data in predicting features of tumour aggressiveness in EC patients investigated for staging purposes.

Our results showed that SUVmax and SUVmean are able to differentiate and predict deep MI, EC risk group, and *p53* expression. Moreover, the metabolic PET parameters MTV and TLG proved to be efficient in predicting deep MI, EC risk group, and LN involvement. Contrarily, none of the imaging parameters demonstrated discriminative ability nor predictive value with respect to the histological subtype. The discriminative ability and predictive power of PET parameters were also compared to those of conventional clinical data known for their association with EC risk, namely patients’ age and BMI. Interestingly, both data showed very poor prognostic value, with only age being predictive of deep MI.

The results reported in the present study corroborate some previously published findings. The available literature reported that SUVmax of primary tumour was significantly higher in high risk patients compared to low risk ones, with sensitivities and specificities in differentiating EC risk groups of 74% and 46%, respectively (supported by the 75% and 61%, respectively, found in the present study) [44]. Similarly. the obtained results agree with some investigations evaluating the relationship between SUV parameters and deep MI [45,46]. According to metabolic parameters, some groups suggested that MTV and TLG might be promising markers for LN involvement [47]; the present work corroborates this hypothesis, as MTV and TLG were the only parameters capable of discriminating and predicting LN metastases. Contrarily, the finding that ^18^F–FDG PET might serve as a predictive tool for *p53* overexpression is novel and of particular interest, this alteration being recognized as a relevant prognostic factor in EC [48]. In fact. the molecular characterization of EC has been fully integrated in the clinical routine for the risk classification of EC patients, as recommended by ESGO/ESTRO/ESP guidelines. This assessment was found to increase the accuracy of the risk classification solely based on the key clinical histological parameters (such as histological subtype, grade, and MI) [25].

One of the major strengths of the present work compared to previous published data is that it relies on the availability of a validation cohort and the possibility of testing acquired knowledge and measured cut-offs. This approach is indeed quite uncommon in conventional statistics; nevertheless, it provided preliminary indication on the actual power of investigated data in patients’ stratification, thus evaluating predictions with the corresponding reference–value and obtaining information on their accuracy, sensitivity, specificity, NPV, and PPV on a validation set. At the same time, this strategy allowed assessment of whether the application of machine learning models in this specific clinical setting might offer additional advantages. ML models are still underrepresented in the field of molecular imaging. According to the little available data, the role of ML models on EC patients has been recently investigated, mainly on MRI images [49,50,51,52], with only anecdotal reports assessing ^18^F–FDG PET scans [53]; of note, in all of these works, ML models were employed to analyze radiomics features. To the best of our knowledge, the present work is the first one assessing the potential of ML models as a valuable tool to analyze conventional PET semiquantitative imaging data (even combined with clinical data) in the primary staging of EC patients. This is particularly significant for both a better physicians’ interpretability of the models’ outcomes and translation into the clinical practice, as the investigated PET parameters are easily assessable on conventional imaging workstations and clinical software.

Some limitations of the present study should, however, be highlighted. Firstly, of the 123 patients included in the study, histological confirmation of the features of EC aggressiveness were not available for all subjects; therefore, the generation of different, smaller sub-populations was necessary for performing the analysis with proper reference standards. Moreover, class imbalances were present, especially in the evaluation of LN involvement. However, to the best of our knowledge, no previous study investigated the efficacy of ^18^F–FDG PET–based ML analysis for predicting deep MI in EC risk group, LN involvement, and *p53* expression using conventional PET parameters extracted from primary EC; thus, further investigations on larger, less unbalanced cohorts are needed to confirm results. Likewise, analyses and ML models implementation were investigated on a monocentric cohort, and validation with external cohorts derived from other Centers is nevertheless required to confirm models’ reproducibility and robustness.

## 5. Conclusions

The present work reports one of the first analyses evaluating the role of machine learning-based classification using ^18^F–FDG PET–derived parameters in predicting features of EC aggressiveness, which are currently assessable only after surgery despite being useful for the most correct treatment in affected patients. Specifically, a signature integrating the most conventional PET parameters (SUVmax, SUVmean, TLG, and MTV) and clinical data (age, BMI) was built aiming at supporting clinicians in the most interpretable and clinically transferable way. From the obtained findings, the described approach showed ability in preoperatively characterizing several features of EC aggressiveness, including EC histological subtype, presence of deep myometrial invasion (MI), presence of lymph–nodal involvement (LN), *p53* expression (wild–type vs. pathological), and overall risk group classification. This demonstrates how advanced PET image analysis based on conventional quantitative parameters and machine learning can support the non-invasive. preoperative stratification and treatment management of EC patients.

## Figures and Tables

**Table 1:** Patient’s characteristics.

Characteristic	Value
Number of patients	123
Mean age. *years (SD)*	65 (10.74)
BMI. *mean (SD)*	27 (5.42)
FIGO stage. *n (%)*: I II III IV	82/123 (66.7%) 12/123 (9.7%) 23/123 (18.7%) 6/123 (4.8%)
Histological subtype. *n (%)*: endometrioid EC non-endometrioid EC	85/123 (69%) 38/123 (31%)
Myometrial Invasion. *n (%)*: <50% >50%	53/115 (46.1%) 62/115 (53.9%)
EC risk group. *n (%)*: low and intermediate risk groups high-intermediate and high-risk groups	43/119 (36.1%) 76/119 (63.9%)
LN involvement. *n (%)* yes no	14/90 (15.6%) 76/90 (84.4%)
*p53* expression. *n (%)* overexpression null	37/51 (72.5%) 14/51 (27.5%)
^18^F-FDG PET finding. *n (%)* pathological uptake non-pathological uptake	119/123 (96.7%) 4/123 (3.3%)

Continuous variables are expressed as mean and standard deviation (SD); dichotomous variables as percentage and ratio. FIGO: International Federation of Obstetrics and Gynecology; EC: endometrial cancer; LN: lymph node; SUV: standardized uptake value; MTV: metabolic tumour volume; TLG: total lesion glycolysis.

**Table 2:** Comparison of the distribution of ^18^F-FDG PET and clinical parameters between the groups defined by the different features of EC aggressiveness.

Feature of EC Aggressiveness	SUVmax	SUVmean	MTV	TLG	Age	BMI
Histological Subtype						
*p*-value	0.080	0.134	0.556	0.389	0.405	0.432
adjusted *p*-value	0.402	0.402	0.556	0.518	0.518	0.518
Myometrial Invasion						
*p*-value	0.001 *	0.007 *	0.002 *	<0.001 *	<0.001 *	0.117
adjusted *p*-value	0.002 *	0.008 *	0.003 *	0.001 *	0.001 *	0.117
EC Risk Group						
*p*-value	<0.001 *	<0.001 *	0.016 *	0.005 *	0.109	0.991
adjusted *p*-value	<0.001 *	<0.001 *	0.024 *	0.010 *	0.131	0.991
LN Involvement						
*p*-value	0.341	0.126	0.001 *	0.003 *	0.929	0.316
adjusted *p*-value	0.409	0.252	0.006 *	0.009 *	0.929	0.409
*p53* Expression						
*p*-value	0.051	0.013 *	0.008 *	0.006 *	0.704	0.499
adjusted *p*-value	0.076	0.026 *	0.024 *	0.024 *	0.704	0.599

Mann–Whitney U test *p*-values adjusted according to the Benjamini-Hochberg correction for multiple testing; EC= endometrial cancer; LN= lymph node; * statistical significance.

**Table 3:** Predictive value of ^18^F-FDG PET and clinical parameters with respect to features of EC aggressiveness on the training set.

Features of EC Aggressiveness	SUVmax	SUVmean	MTV	TLG	AGE	BMI
**Histological subtype** AUC 95% CI Optimal cut–off Sensitivity Specificity	0.421 0.29–0.55 / / /	0.458 0.33–0.59 / / /	0.473 0.35–0.60 / / /	0.469 0.35–0.59 / / /	0.418 0.29–0.55 / / /	0.566 0.45–0.69 / / /
**Myometrial invasion** AUC 95% CI Optimal cut–off Sensitivity Specificity	0.712 0.60–0.81 * 14.850 74% 62%	0.676 0.56–0.79 * 8.556 72% 60%	0.681 0.57–0.79 * 10.980 58% 76%	0.711 0.60–0.81 * 96.125 64% 71%	0.749 0.64–0.85 * 66.449 70% 73%	0.564 0.44–0.69 / / /
**EC risk group** AUC 95% CI Optimal cut–off Sensitivity Specificity	0.671 0.55–0.79 * 14.188 75% 61%	0.671 0.55–0.79 * 9.694 63% 71%	0.648 0.52–0.77 * 5.133 78% 52%	0.652 0.53–0.77 * 96.125 56% 68%	0.602 0.48–0.72 / / /	0.482 0.35–0.61 / / /
**LN involvement** AUC 95% CI Optimal cut–off Sensitivity Specificity	0.577 0.40–0.74 / / /	0.611 0.42–0.78 / / /	0.815 0.70–0.91 * 10.980 90% 71%	0.794 0.64–0.93 * 251.823 60% 90%	0.523 0.29–0.75 / / /	0.579 0.38–0.76 / / /
***p53* expression** AUC 95% CI Optimal cut–off Sensitivity Specificity	0.734 0.52–0.93 * 16.286 69% 75%	0.830 0.67–0.96 * 9.536 81% 75%	0.691 0.41–0.96 / / /	0.723 0.43–0.99 / / /	0.533 0.31–0.74 / / /	0.455 0.23–0.68 / / /

AUC = area under the curve; CI = confidence interval; EC = endometrial cancer; LN = lymph node; * statistical significance. SUV = standardized uptake value; MTV = metabolic tumour volume; TLG = total lesion glycolysis; BMI = body mass index.

**Table 4:** Predictive performances of ^18^F–FDG PET and clinical parameters. alone and combined in Random Forest Classifiers. on the validation set.

Features of EC Aggressiveness	SUVmax	SUVmean	MTV	TLG	Age	RFC
**Myometrial invasion**						
	th: 14.85	th: 8.56	th: 10.98	th: 96.13	th: 66.45	RFC_MI_
Accuracy	52%	52%	52%	61%	48%	87%
Sensitivity	67%	67%	42%	58%	42%	100%
Specificity	36%	36%	64%	64%	55%	73%
PPV	53%	53%	56%	64%	50%	80%
NPV	50%	50%	50%	58%	46%	100%
**EC risk group**						
	th: 14.19	th: 9.69	th: 5.13	th: 96.13		RFC_RG_
Accuracy	71%	67%	54%	62%	/	79%
Sensitivity	75%	50%	67%	58%	/	92%
Specificity	67%	83%	42%	67%	/	67%
PPV	69%	75%	53%	64%	/	73%
NPV	73%	62%	56%	62%	/	89%
**LN involvement**						
			th: 10.98	th: 251.82		RFC_LN_
Accuracy	/	/	61%	72%	/	83%
Sensitivity	/	/	50%	25%	/	25%
Specificity	/	/	64%	86%	/	100%
PPV	/	/	29%	33%	/	100%
NPV	/	/	82%	80%	/	82%
***p53* expression**						
	th: 16.29	th: 9.54				RFC_p53_
Accuracy	45%	45%	/	/	/	73%
Sensitivity	20%	40%	/	/	/	100%
Specificity	67%	50%	/	/	/	50%
PPV	33%	40%	/	/	/	63%
NPV	50%	50%	/	/	/	100%

For each PET parameter. Cut-offs were derived from ROC curves using the train set, and used as thresholds (th) to classify patients from the validation set; RFC: Random Forest Classifier; MI: myometrial invasion; RG: risk group; LN: lymph node.

**Table 5:** Hyperparameters selected for the implementation of the four Random Forest Classifier (RFC) algorithms.

Hyperparameters	Myometrial Invasion	EC Risk Group	Lymph Node Involvement	*p53* Expression
n_estimators	24	22	2	5
max_depth	None	None	None	None
min_samples_split	5	2	2	2
min_samples_leaf	2	4	1	1
max_features	auto	auto	auto	Auto
bootstrap	True	True	True	True
class_weight	1:1	1:2	1:1.5	1:1

Hyperparameters are defined according to the Scikit–learn library [39].

## Data Availability

All data needed to replicate our analyses are available upon request from the corresponding author.
